# National Insurance and the Voluntary Hospitals

**Published:** 1912-02-24

**Authors:** 


### NATIONAL INSURANCE AND THE VOLUNTARY HOSPITALS.

### SPEECH BY MR. WORTHINGTON EVANS, M.P.

A largely attended meeting, under the auspices of the Incorporated Society of Hospital Officers, was held in the Great Hall of St. Bartholomew's Hospital on Friday, February 16. Mr. Kershaw, the President of the Association, was in the chair, and in the course of his remarks explained the purpose of the meeting to be neither to praise nor to condemn the new Insurance Act, but to inquire what were its possibilities.

Mr. Worthington Evans, M.P., then addressed the meeting. He disclaimed any special knowledge of the management of hospitals, though he had been on the committee of a voluntary hospital. But he had been busy trying to understand what the National Insurance Act was. The two clauses specially concerning hospitals were Nos. 12 and 21. If an agreement for payment were made by a hospital or convalescent home with the friendly society in which an insured person is, then all or part of the sickness and disablement benefit to which that person may be entitled could be paid over to the hospital or convalescent home, provided such person had no dependents. That was the first possible source of income of hospitals and convalescent homes under the Act. If the patient had dependents, then those dependents received the sick benefit while the patient remained in hospital. Dependents, however, were not defined in the Act. In paying hospitals the mere fact that the dependents were to some extent provided for under the Insurance Act would free other funds which the insured person might have, and would enable him to make some payment to the hospital. Another source of income was the 30s. maternity benefit payable to the "doubly-insured woman"—a woman who was herself employed and was at the same time the wife of an insured man. She, on confinement, was entitled to 7s. 6d. per week for four weeks as sick pay, and, by virtue of being the wife of an insured man, she was entitled to 30s. In that event the 30s. *may*, if the friendly society wish, be paid over to the hospital. There would not be, relatively, many "doubly-insured" women. There was nothing to be received from the large class known as deposit contributors—namely, those who had such health that they were unable to get into a friendly society by the medical test, and had to have their contributions placed to their credit in the post office. That class was likely to come more frequently to the hospital.

### No Hospital Benefit.

Under Section 21 there was a possible source of income—namely, that the friendly societies and county committees may subscribe to the hospitals, but there was no compulsion. A letter had been written to him asking whether the Treasury and County Councils had power to supplement the contributions made by friendly societies to hospitals. They had no such power under the Act. But the Treasury and the Councils might, if they so agreed, contribute, out of the rates and taxes, a certain addition to the medical expenses. The new Act did not include any hospital benefit; there was a sickness benefit, a disablement benefit, a maternity benefit, a sanatorium benefit, but there would be no contribution for diseases other than tuberculosis or kindred complaints. The additional cost of the extra patients which hospitals would have to deal with if every man who had paid weekly contributions had the right to hospital treatment would be very great indeed. There would be trouble with the medical staffs, who, under those conditions, would probably require some sort of payment. As to how hospitals were likely to be affected under the Act, one thing was certain: that for every male and female employee of hospitals those hospitals must make a weekly contribution. In a hospital of 250 beds there would probably be employed 32 males and 128 females—a total of 160, and the annual tax payable for them would be £104; and, in addition, the male and female employees would contribute together £120, including £52 a year for medical benefit. It seemed odd that it should have been thought necessary to make employees of hospitals—surrounded by all the appurtenances of health, and entitled, as part of their service, to treatment without payment—pay 1½d. per week each towards the payment for medical benefits for treatment which, hitherto, they had received for nothing. Dealing with matters of a more speculative nature, he thought the effect of the Act would be somewhat to diminish the attendance at the out-patient departments.

Employed people would have the right to go to their doctor for treatment; and some of them, not having previously had that right, had drifted to out-patient departments. But, on the other hand, the deposit-contributor, the man in worse health, who was least regularly employed, would frequently require medical benefit, and to that class the Act would make but little difference.

He believed there would be a definite increase of in-patients. The first reason was a wholly estimable one. If a very widespread system of medical treatment were set up, brought within the reach of large numbers who had, perhaps, hesitated to go to their doctor before, a large number of cases would be discovered who required and deserved treatment in hospital, and who otherwise might have gone on to death. Another reason was, that human nature was human nature, even in doctors. If one compelled a doctor to take in, by contract, large numbers of patients, he would discover who were the troublesome ones; that doctor might fall into the same temptation as the German doctors had, and shift such patient off to the hospital.

### The Financial Prospect.

Mr. Evans next proceeded to the question of finance. He had seen suggestions in the Press that the Act would affect the finances of hospitals detrimentally, but he doubted this. He thought the income at present derived from workmen's contributions would be detrimentally affected by the measure. The Royal Victoria Hospital, Newcastle-on-Tyne, had an income of £33,000, of which the sum of £17,000 was raised by workmen's contributions. In 1911, out of £954 received by the Essex County Hospital, Colchester, £345 was from workmen. But when such workmen were asked to contribute 4d. per week for sickness insurance, that must have an adverse effect upon their contributions. With regard to the effect of the Act on special hospitals and convalescent homes, including paying hospitals, most of the convalescent homes and paying hospitals were not run by voluntary contributions, but were supported by them. It was argued that those who went into paying hospitals would not receive anything under the Act which would enable them to pay their contributions to the hospitals, and that thus the hospitals were likely to suffer. As an instance of a paying hospital, he took the Clapham Maternity Hospital, with a total income of £1,953. Any inmate of that hospital, if she had dependents, would not be entitled to the maternity benefit. The patients' payments were £725, or 38 per cent.

of the income. The practice was for the patients to pay in advance for the benefits to be received there. If the woman entering there had dependents, she would not receive maternity benefit, and so that could not be transferred to the hospital. A woman was entitled to maternity benefit if she were the wife of an insured man who was in benefit. But all the children in the family were the dependents on the man, not on the woman. He advised the kind of hospital of which he was speaking quickly to make arrangements with approved societies which would administer those benefits; then, instead of the woman paying contributions in advance, she would be entitled to go into that hospital, and the hospital would be entitled to receive the 30s. which was payable in respect of her confinement. The deposit-contributor class would seldom have enough to their credit to give them any maternity benefit. If this class had anything to do with hospitals, the hospitals would make provision : the Act did not help at all.

With regard to the formation of societies by those interested in hospitals, for the nurses and other employees of hospitals, he believed most of the men engaged in hospitals were in some society : at any rate, before an attempt were made to form a separate society for men inquiry on the point should be made. But for women there were very few such friendly societies worth speaking of; and they should have such a society, with a total membership of not less than 5,000, for then they would be entitled to the whole of the surplus which there might be. While at work in hospitals, nurses, if they fell sick, would be looked after there, and they would need the 7s. 6d. per week more later in life, when they would probably be less able to resist sickness.

Mr. Kershaw said that if one took the hospitals in groups, the general and the special, in the former the income from subscriptions was only 11 per cent.; the donations were 19 per cent. of the total. In the King's Fund it was 12 per cent., and in the Saturday Fund 1 per cent. to general hospitals, 3 per cent. to special hospitals. The main income was from investments : 43 per cent. to general, and 15 per cent. to special hospitals. He thought, in opposition to Mr. Evans, that the attendance as out-patients would be increased, because the moment there was any doubt about what was the matter with a patient, he would go to the hospital as a consultation centre between the hospital and the medical man. Then disease would be caught at the beginning, to the benefit of the whole community.

### SOME QUESTIONS AND ANSWERS.

Mr. Albey (Charing Cross Hospital) desired more light on what was meant by the words, dealing with sanatorium treatment, "or other institutions," and "or such other diseases." He asked particularly in conjunction with Section 64, dealing with supplementary provisions, which conferred great powers on Insurance Committees and County Councils to erect buildings and deal with them in every way.

Mr. Evans replied that, with respect to "other institutions," nothing could be said beyond that conveyed by the words in the Act. In the debate in the House it was pointed out that medical opinion was not yet finally fixed as to the desirability of spending much money on bricks and mortar in building sanatoria, as there were alternative methods of treatment. But it was really premature to discuss that now.

Mr. Burleigh (Hospital for Epilepsy, etc.) said that once an insured patient came into benefit, he belonged, body and soul, to his insurance company. He suggested that when the Insurance Commissioners had a large number of patients who might be termed "work-shy," or malingerers, in order to get them out of benefit steps must be taken to provide appropriate treatment for them.

Dr. F. J. Poynton, speaking as a doctor on the staff of a hospital, said that at the present time there was some difficulty between the doctors and the working of the Act, and probably no one knew what exactly would happen; but a considerable number of doctors on the staffs of those hospitals had signed a pledge not to work under the Act. If such doctor refused to serve on panels, he was anxious to know what the position of the doctors on the large voluntary hospitals would be. He took it that, as a voluntary worker in a voluntary institution, such a one would not be, technically, working under the Act. It was certain that the poor people—Act or no Act—must be attended to; but supposing certain contributions were made to the hospital from insured people, then, as voluntary workers in such institution, would those doctors referred to be considered as working under the Act? Possibly the doctors might have to ask of their lay committees some patience and a consideration of the position until it had righted itself.

Mr. Evans replied that the fact that such a doctor was a voluntary worker almost defined his position. Even a contribution to a hospital by a friendly society was a mere grant; it gave no right to the grantor whatever; and as there was no right, there was no contract. Such a doctor would not place himself in opposition to his pledge.

Mr. McAdam Eccles, M.S., detailed the various classes of patients with whom general hospitals dealt, and expressed the opinion that there would be no diminution of numbers in either the in-patient or out-patient departments; and the pressure on the beds of most hospitals was already great, so that patients were sometimes sent out before they were finally cured. Directly contract practice was set up, however humane the doctor might be, the relationship between doctor and patient was altered, and in the case of troublesome patients the question arose, how could they be got rid of? The hospital was the means, those working in hospitals knew it. Nothing had been said that evening about medical benefit. He wanted to know why those in voluntary hospitals should be, as it were, forced to treat patients for whom the State expressed the intention to provide the money for treatment. He deprecated anything like control of hospitals from the outside; he had seen the State hospitals of Germany, and was struck by their parsimonious economy—a State hospital in Germany of the size of St. Bartholomew's, with 600 beds, had 50 nurses, whereas St. Bartholomew's had 344.

Mr. Jennings asked whether, by some amending Act, provision could be made for giving a payment to hospitals for services rendered. In the Act there was provision for supervising and inspecting approved societies.

Mr. Evans replied that State inspection was closely related to State control; it was not a question of principle so much as of degree.

Mr. Greaves (Sussex Eye Hospital) said the Governors of his hospital had passed a rule that gave them power to refuse free treatment to any who by Act of Parliament were entitled thereby to medical treatment. That was merely to safeguard themselves against an overplus of patients.

Sir John Young spoke decisively against anything like State control of hospitals, and complained of the hurried way in which such a far-reaching measure was passed into law.

